# Malnutrition and Climate in Niger: Findings from Climate Indices and Crop Yield Simulations

**DOI:** 10.3390/ijerph22040551

**Published:** 2025-04-02

**Authors:** Benjamin Sultan, Aurélien Barriquault, Audrey Brouillet, Jérémy Lavarenne, Montira Pongsiri

**Affiliations:** 1 ESPACE-DEV, University Montpellier, IRD, University Guyane, University Reunion, University Antilles, University Avignon, Maison de la Télédétection, 500 Rue Jean-François Breton, 34093 Montpellier, France; audrey.brouillet@ird.fr; 2 Save The Children, London WC2H 7HH, UK; aurelien.barriquault@savethechildren.org (A.B.); mpongsiri@gmail.com (M.P.); 3 CIRAD, UMR TETIS, University Montpellier, AgroParisTech, CIRAD, CNRS, INRAE, 34398 Montpellier, France; jeremy.lavarenne@cirad.fr

**Keywords:** climate, malnutrition, Africa, drought, crop model

## Abstract

Malnutrition, particularly its impact on child morbidity and mortality, is one of the top five health effects of climate change. However, quantifying the portion of malnutrition attributed to climate remains challenging due to various confounding factors. This study examines the relationship between climate and acute malnutrition in Niger, a country highly vulnerable to climate change and disasters. Since climate’s effect on malnutrition is indirect, mediated by crop production, we combine rainfall data from TAMSAT satellite estimates with the SARRA-O crop model, which simulates the impact of rainfall variability on crop yields. Our analysis reveals a significant correlation between malnutrition and both rainfall and crop production from the previous year, but not within the same year. The strongest correlation (R = −0.72) was found with the previous year’s crop production. No significant links were found with temperature or intra-seasonal rainfall indices, like the start or duration of the rainy season. Although national correlations between global malnutrition, rainfall, and crop yields were stronger, they were weaker or absent at the regional level and, for Severe Acute Malnutrition crises, are less likely driven by climate variability. However, the one-year lag in the correlation allows for the prediction of future food crises, providing an opportunity to implement early intervention measures.

## 1. Introduction

Africa is amongst the most food-insecure regions globally, with approximately 20% of the population undernourished [1]. Factors such as the war in Ukraine, the aftermath of the COVID-19 pandemic, armed conflicts, currency devaluations, inflation, and climate extremes are derailing progress towards achieving Sustainable Development Goal 2 (SDG) on food security and nutrition. Climate change exacerbates these challenges, pushing food systems to their limits as malnutrition and climate change directly relate to SDG 2 (Zero Hunger), SDG 3 (Good Health and Well-being), SDG 11 (Sustainable Cities and Communities), and SDG 13 (Climate Action). Between 2000 and 2022, climate change caused USD 11.5 billion in crop and livestock losses in the Sahel and Greater Horn of Africa, contributed to 12,000 deaths from droughts and floods, and affected nearly 149 million people in the region [2]. In West Africa, Sultan et al. [3] identified historical global warming as a driver of crop production losses, while future crop yield reductions are projected in various studies. For example, Alvar-Beltrán et al. [4] applied a climate crop modelling approach in Niger using AquaCrop and predicted yield reductions of up to −50% for major staple crops, such as millet and sorghum, by the end of the century (2060–80). Similarly, and under different climate and socioeconomic scenarios, Iizumi et al. [5] projected that Sudan’s share of domestic production could drop from 16.0% to 4.5–12.2% by 2050, even with adaptation measures. In response, international organizations such as FAO, AUC, ECA, and WFP call for sustainable solutions to strengthen food security, increase agricultural productivity, diversify diets, and build community resilience [1].

The situation is particularly dire in West and Central Africa, where the World Food Program [6] estimated a fourfold increase in severe food insecurity over the past five years. In Western African countries, high food prices and fragile food systems have resulted in inadequate diets, limited access to basic services, and poor care and hygiene practices, further exacerbating acute malnutrition in children under five, adolescents, and pregnant women, already driven by conflict, climate shocks, and disease outbreaks. In this region, malnutrition affects 16.7 million children under five, and two-thirds of households cannot afford a healthy diet. Malnutrition rates, through both wasting and stunting, are particularly severe in Nigeria [1]. There, and in many other Sub-Saharan countries, these malnutrition rates are associated with risk factors such as mother education, poverty level, diarrhea incidence, low birth rate, and many others [7]. A nutrition causal analysis conducted by FEWS NET in Niger’s Maradi and Zinder regions reported a significant seasonal variation in the prevalence of child wasting in the agropastoral and agricultural livelihood zones [8], and highlighted child morbidity as a consistent risk factor.

While malnutrition, and especially its impact on child morbidity and mortality, is considered to be one of the five most important adverse health impacts of climate change, quantifying its direct attribution to climate change remains challenging due to complex confounding factors [9]. Nevertheless, Phalkey et al. [9] found significant but variable associations between weather variables (e.g., rainfall; extreme weather events, such as floods/droughts; seasonality; and temperature) and child stunting in 12 of the 15 studies reviewed. In low-income settings, high levels of child malnutrition are often observed in drought-affected populations, but the extent of drought’s direct impact remains unclear and context-specific. In Low and Middle Countries, a systematic review conducted in various Sub-Saharan African countries observed a significant correlation between climate variables, temperature, rainfall, and drought, and at least one undernutrition parameter in 19 out of 22 studies [10]. Findings on the relationship between climate variability and acute malnutrition risk in Sub-Saharan African countries reported that precipitation deficits and high temperatures are associated with reduced child weight-for-height [11]. In Burkina Faso, evidence shows that short-term exposure to high temperatures, particular during crop seasons and droughts, has a negative impact on child health, along with an increased risk of infant and child mortality and incidence of wasting [12]. In Ethiopia, Malawi, Mali, Niger, and Tanzania, food insecurity increases by 5–20 percentage points with each drought or flood event [13]. In West Africa, higher long-term average temperatures are more negatively associated with dietary diversity than even household poverty [14]. Spatial analyses in Mali and Kenya further link livelihoods and measures of malnutrition to weather variables and stunting [15,16]. In Nigeria, rising temperatures directly exacerbate childhood malnutrition—especially in rural areas—while precipitation changes have an indirect effect, thus threatening to reverse years of progress in reducing child undernutrition [17]. In West Africa, climate variability and armed conflict notably heightened the risk of childhood diarrhea and malnutrition, with children exposed to the Boko Haram insurgency in Northeast Nigeria experiencing significantly higher rates of wasting and diarrhea [18]. In many conflict- and climate-prone contexts, near real-time data and modeling can be used to anticipate malnutrition crises and inform proactive, location-specific interventions for preventing acute malnutrition in children [19]. In Niger, climate-driven disruptions to livestock production—particularly reduced access to milk—have contributed to persistently high rates of acute malnutrition among children under five [20]. In Niger, recurrent droughts and floods—particularly evident during the severe crises of 2005 and 2010—fuel chronic food insecurity, undermining maternal and neonatal nutrition efforts and driving persistently high rates of child malnutrition despite government-led, multisectoral resilience measures [21].

This study examines the link between climate and acute malnutrition in Niger, identified as the world’s most climate-vulnerable country (in terms of exposure and sensitivity to the adverse effects of disasters and climate change) by the Notre Dame Global Adaptation Initiative (ND-GAIN; https://gain.nd.edu/our-work/country-index/rankings/; accessed on 26 March 2025). The interaction between the level of acute malnutrition and acute events that can be explained by year-to-year food insecurity events is also explored. We assess statistical relationships at multiple scales between malnutrition indicators and climate indicators from satellite and in situ sources. Recognizing that the relationship between climate and malnutrition is mediated by crop production responses to climate, we integrate rainfall indices with crop model outputs to simulate the impacts of rainfall variability on agricultural yields and crop production.

## 2. Data and Methods

### 2.1. Geographical Area

Niger is a large landlocked country with a population of about 21.5 million people (2021), and two-thirds of it is located within the Sahara Desert [22]. The population is mostly concentrated along the Niger River in the southwest and along its southern border (with Nigeria). The major economic activity of Niger lies primarily in agriculture, livestock, and also informal trade and production. The rainy season, which typically lasts from July to September, receives between 300 and 750 mm of rainfall. Climatically, the country has experienced declining average rainfall, desertification, recurring droughts, and deforestation. The impact of such human activities has led to increasing risks to agriculture (farmer-led irrigated agriculture), with consequent widespread malnutrition in Niger.

### 2.2. Nutrition Data and Socioeconomic Indices

**Figure 1:**
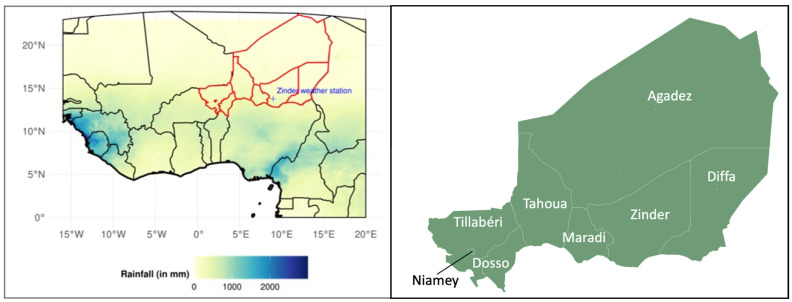
Spatial distribution of averaged annual June-to-September cumulated rainfall over 1983–2022 from TAMSAT (in mm) and administrative map of Niger.

The prevalence rates of Global Acute Malnutrition (GAM) and Severe Acute Malnutrition (SAM) among children aged 6 to 59 months were used as nutrition metrics. These rates are defined based on the weight-for-height (W/H) index. GAM corresponds to a W/H Z-score below −2 standard deviations compared to a reference population, while SAM is defined as a W/H Z-score below −3 standard deviations. The classification of nutritional situations follows the Integrated Phase Classification (IPC) standards, with a GAM rate ≥15% indicating a phase-four (critical/emergency) level. Historical data on GAM and SAM prevalence rates were compiled from multiple sources. Data from 2005 to 2022 were obtained through the Standardized Monitoring and Assessment of Relief and Transition (SMART) surveys, while earlier data from 1992 to 2000 were sourced from Demographic and Health Surveys (DHSs) and Multiple Indicator Cluster Surveys (MICSs). These datasets were accessed via the National Institute of Statistics (INS), the WHO/UNICEF databases, and related sources. Data gaps were noted for the years 1993–1995, 1997, 1999, and 2001–2004. Data were collected at the administrative level (8 regions in Figure 1).

A set of 53 socioeconomic indicators from the World Development Indicators database was used to assess confounding factors in the correlation analysis between climate, crop production, and malnutrition. These indicators are available at the national level and were extracted from 1990 to 2022. A complete list of the indicators, along with metadata, is provided in Supplementary Table S1.

### 2.3. Food Crisis Classification

In Niger, a food crisis is declared based on several factors, including food availability, access, utilization, and stability. The Integrated Food Security Phase Classification (IPC) is used to assess and classify the severity of food insecurity. The IPC framework classifies the severity of food insecurity into five phases: Minimal, Stressed, Crisis, Emergency, and Famine. A food crisis is declared when a significant portion of the population is in the Crisis phase (IPC Phase 3 or more). The IPC analysis is usually conducted twice a year as part of the regional Cadre Harmonisé and the Food Crisis Prevention Network (RPCA) in Western African countries (The Cadre Harmonisé and IPC collaboration). The analysis included the different years where a food crisis was declared by the Government of Niger; NGOs; and secondary analyses, including 2005 [23], 2010 [24], 2012 [25], and 2022 [26].

### 2.4. Climate Data and Indices

#### 2.4.1. The TAMSAT Rainfall Estimates

We analyzed rainfall data from the The Tropical Applications of Meteorology using SATellite data and ground-based observations (hereafter TAMSAT; [27,28]). This dataset provides daily rainfall estimates over Africa at a 0.0375° spatial resolution from 1983 to the present. TAMSAT offers one of the highest spatial resolution rainfall datasets and incorporates data from numerous African national meteorological and hydrological centers, thereby expanding the in situ dataset for assimilation. The main advantage of TAMSAT is that it provides updates almost immediately, making it useful for monitoring and early warning systems. One of the organizations that uses TAMSAT is Niger’s AGRHYMET, which produces a Monitoring Bulletin for the agro-pastoral season in the Sahel and West Africa every ten days during the rainy season, based on TAMSAT data (see, for instance, https://agrhymet.cilss.int/2024/08/30/bulletin-de-suivi-de-la-campagne-agropastorale-au-sahel-et-en-afrique-de-louest-situation-au-31-juillet-2024/; accessed on 26 March 2025). However, as it may be less accurate than other products (e.g., CHIRPS) over West Africa due to sparse gauge network [29], we compared the rainfall estimation to station data. To provide localized and relevant information in Niger, all time series and statistics presented in this paper are based on data averaged at the administrative-region level. The 8 regions of Niger are shown in Figure 1. Since the Niamey region is smaller than one pixel, it is excluded from the analysis.

#### 2.4.2. The Zinder Meteorological Station

**Figure 2:**
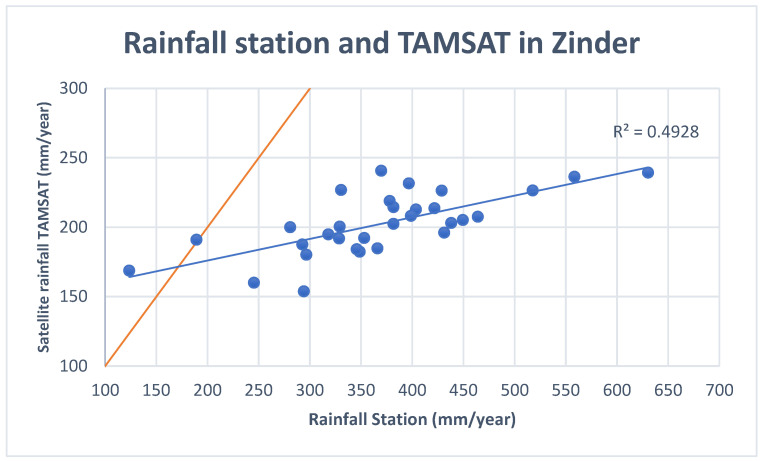
Comparisons between TAMSAT annual rainfall estimates and rain gauge data in Zinder. TAMSAT Rainfall data were aggregated over the Zinder region area between 1991 and 2021. Rain gauge data were collected by the Meteorological Service in Niger.

In addition to satellite rainfall estimates, we collected rain gauge data from the Zinder station (Lat 13.779, lon 8.984, Elevation: 1516 ft) for the period from 1991 to 2021. These data were provided by the Meteorological Service of Niger. To assess the accuracy of TAMSAT rainfall estimates, we compared the annual mean rainfall over the Zinder region with the rain gauge data. TAMSAT rainfall estimates closely align with observations at the Zinder station, with an R^2^ = 0.49, although TAMSAT significantly underestimates annual rainfall (Figure 2). This bias may arise from comparing a single station value (the Zinder station meteorological station) to TAMSAT rainfall averaged over the entire Zinder region, which covers a large area of 155 778 km^2^. Additionally, inherent biases in the satellite product may contribute to inaccuracies in reproducing rainfall in the region. Numerous studies have emphasized the difficulties encountered by satellite rainfall estimates in accurately capturing rainfall variability in West Africa [29,30,31,32,33]. This is primarily due to the significantly lower gauge density in this region when compared to other regions of the world. This sparse gauge network could affect the reliability of products such as TAMSAT, which rely on rain gauge data for calibration. While this limitation may influence the accuracy of the relationships between malnutrition and rainfall, satellite rainfall estimates remain critical for research due to the limited availability of rain gauge data to the scientific community and the public accessibility of most satellite products.

#### 2.4.3. The Rainfall Indices

Several rainfall metrics were calculated to investigate possible correlations between rainfall patterns/extremes and malnutrition (Table 1). These metrics belong to a longer list of user-relevant indices for agriculture from the AMMA-2050 project and have been shown to be strongly related to crop production [34]. First, we examined the June-to-September cumulative rainfall amount to assess the interannual variability of total seasonal rainfall. We also analyzed the rainy season onset and corresponding cessation. These two metrics were calculated using the method of Liebmann and Marengo [35], as described in Bombardi et al. [36], which relies on accumulated precipitation anomalies. For each year in the study period, daily accumulated anomalies were computed and smoothed using a 15-day moving window to eliminate synoptic variability. The rainy season was defined as the longest consecutive period when this smoothed difference in daily accumulated anomalies remained positive (above 0). The onset (cessation) of the season was determined as the first (last) day of this rainy period. The duration of the rainy season is derived from the number of the days between the onset and the cessation of the rain. Lastly, we calculated the number of dry spells, defined as consecutive dry days (daily rainfall < 1 mm) within the rainy period.

#### 2.4.4. The Temperature Indices

We included temperature-related variables (e.g., heatwaves, mean temperature trends, and extreme temperature events) to provide a more comprehensive climate–malnutrition link (Table 1). Temperatures data were extracted from the high-resolution global dataset of meteorological forcings for land surface modeling [37]. We computed different temperature indices recommended by the WMO and the WCRP Expert Team on Climate Change Detection and Indices [38] from the simplest ones (mean surface temperature, and minimum and maximum temperatures) to more complex indices, representing extreme day and night temperatures and heatwave duration (warm spell-duration index).

**Table 1:** Synthesis of climate metrics analyzed in this study (with corresponding acronyms, units, and references).

Climate Indice	Short Name	Units	References
Cumulated JJAS Rainfall	JJAS Rainfall	mm/season	-
Onset of the rain	Onset	Day of the year	Liebmann and Marengo (2001) [35], Bombardi et al. (2019) [36]
Cessation of the rain	Cessation	Day of the year	Liebmann and Marengo (2001) [35], Bombardi et al. (2019) [36]
Duration of the rainy season	Duration	Days	Liebmann and Marengo (2001) [35], Bombardi et al. (2019) [36]
Number of dry spells	Dry Spells	Days/season	
Mean surface temperature	Tmean	°C	
Minimum temperature	Tmin	°C	
Maximum temperature	Tmax	°C	
Percentage of days when TMax > 90th percentile	Tx90p	%	Zhang et al. (2011) [38]
Percentage of days when TMin > 90th percentile	Tn90p	%	Zhang et al. (2011) [38]
Warm spell duration index: annual count of days with at least 6 consecutive days when TMax > 90th percentile	Wsdi	Days	Zhang et al. (2011) [38]

### 2.5. The Crop Model

In this study, we used the SARRA-O crop model to investigate variations in attainable yield of millet in Niger. SARRA-O (DOI:10.5281/zenodo.11091698) is a spatialized adaptation of the SARRA-H crop simulation model [39]. It enables gridded simulations over spatial extents through the direct use of georeferenced raster datasets such as TAMSAT products. Based on the simple formalisms of SARRA-H, SARRA-O falls into the crop model categories of “simple (descriptive) radiation use efficiency approach” for light utilization and biomass growth, and “fixed harvest index” and “partitioning during reproductive stages” for yield formation [40]. SARRA-O simulates plant emergence and growth for annual crops on a daily time step. It uses thermal time thresholds since emergence to account for growth stages, which in turn govern the simulation of plant growth processes, primarily biomass production and allocation between organs. A bucket-type water balance is computed daily, incorporating soil water storage capacity through shallow and deep reservoirs and simulated rooting depth. Overall, this deterministic hypothesizes that crop productivity is defined and limited by accumulated hydrological constraints throughout growth cycle. The simulation grid aligns seamlessly with soil water retention capacity, climate data reanalysis, and satellite rainfall-estimate gridded datasets, derived from ISRIC SoilGrids maps [41], AgERA5 [42], and TAMSAT products [28], respectively. The model’s initial resolution is based on satellite rainfall estimates, ensuring an accurate reflection of rainfall on crop yield.

Sultan et al. [3] validated the crop model by comparing the annual yield time series reported by the FAO, with simulated yields for millet and sorghum across several West African countries, spanning the period of 1961–2012. The country-level comparisons show that the model effectively simulates yield anomalies for both crops, with significant correlations in Niger, Mali, and Burkina Faso—three major crop-producing nations in the region. In Niger, the correlation coefficients range from R = 0.59 for millet to R = 0.64 for sorghum. However, the model struggles to capture linear increasing trends because it does not account for technological advancements or the CO_2_ fertilization effect. Additionally, it tends to overestimate the average yield, a common issue in crop models developed in controlled environments that overlook non-climatic factors like pest and disease influences. In this study, we assume that the positive mean bias in crop production remains relatively stable year to year, allowing us to compare simulated (climate-driven) yield variability with malnutrition indices. We also checked that the correlations between crop yield and malnutrition remain robust even after removing linear trends.

For this study, we performed simulations with SARRA-O (v1.11 _hotfix_20230331) across Niger, using typical crop parameters for millet, calibrated and validated during previous experiments (see list of used parameters in Supplementary Table S2). The simulations covered the period of 1989–2022, using AgERA5 weather data and TAMSAT satellite rainfall estimates. SARRA-O was used to simulate the annual attainable millet yield (subject to water stress but excluding heat, wind, or fertilizer stress). The gridded simulation results were aggregated at the ADM1 administrative level (regions), and averaged values were computed without the use of cropland masks.

### 2.6. The Correlation Coefficients

We computed correlation coefficients between malnutrition (GAM and SAM) and rainfall indices and crop yields between 1991 and 2022. Correlations were computed at the regional level and at the national level by averaging data at the national level. Caution should be exercised in interpreting the national mean of GAM and SAM, as it is based on a mean of regional values and does not take into account disparities in population density between the regions. This choice was made in order to not allow a limited geographical area with a high population density (near Niamey), which could introduce a bias in the calculation of the correlation with national rainfall, to have too much influence. Calculating several correlations on the same time series of GAM and SAM increases the risk of a type I error, i.e., to erroneously conclude the presence of a significant correlation. The chances to reject the null hypothesis when it is true (i.e., detecting a significant relationship when the two time series are independent) increase with the number of tests performed. To avoid this, the level of statistical significance of correlation coefficients is adjusted using the Bonferroni correction [43]. We fixed the error rate at 5% (alpha = 0.05), and now the threshold level of significance is based on an error rate at alpha/n, where n is the number of tests performed.

## 3. Results

### 3.1. Evolution of Nutrition Indicators in Niger

**Figure 3:**
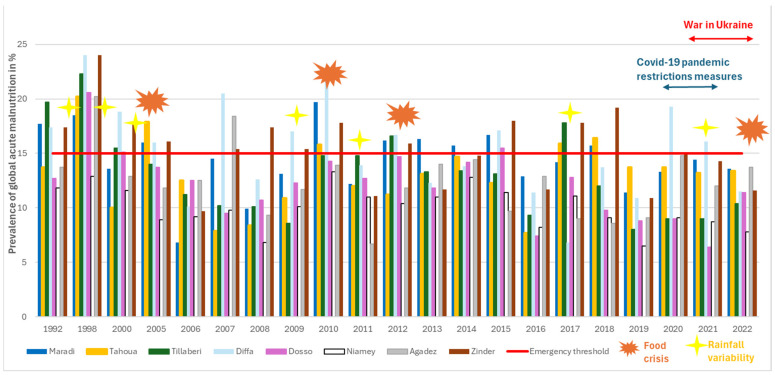
Trends in Global Acute Malnutrition prevalence rate across 8 regions in Niger from 1992 to 2022.

The trend in the Global Acute Malnutrition (GAM) prevalence rates across Niger’s regions from 1992 to 2022, as illustrated in Figure 3**,** shows that seven out of the eight regions reached the 15% emergency threshold in the year of 1998. From 2005 to 2022, the emergency limit for GAM was reached every 2–3 years in 3–4 of the 8 regions until 2018. However, from 2019 to 2022, there was a decrease in the number of cases of GAM, with only one or no regions going beyond the emergency limit during this period. Half of the regions were categorized in the emergency phase in different years affected by food crises (2005, 2010, and 2012), except for the food crisis declared in 2022, where none of the regions reached the emergency threshold for GAM. Various shocks preceded these crises, including rainfall deficit (quantity or poor rainfall distribution) in the prior years (1997, 2004, 2009, and 2011) [44], locust invasions (2004–2005 and 2012), and significant reductions in cereal production (1998, 2005, 2009, 2011, and 2021), as well as high food prices [45]. The regions of Zinder, Maradi, and Diffa were most frequently affected by high GAM prevalence, exceeding the emergency threshold 12, 10, and 9 times, respectively, over the 21 years of data points. Since 2019, Zinder and Maradi have shown a similar trend of GAM reduction below the emergency threshold but remain in the “serious” phase classification (>10%). In contrast, Diffa experienced a significant increase in GAM rates in 2020 and 2021.

**Figure 4:**
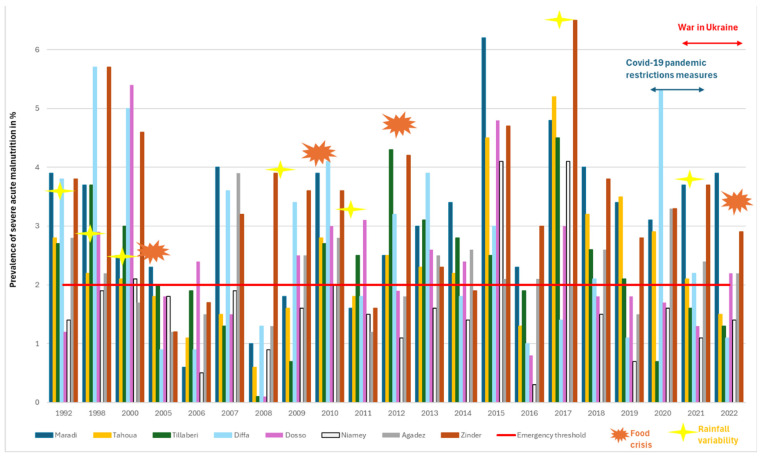
Trends in Severe Acute Malnutrition prevalence rate across 8 regions in Niger from 1992 to 2022.

Figure 4 illustrates the trends in Severe Acute Malnutrition (SAM) prevalence rates. The emergency threshold for SAM was reached 17 times out of the 21 years (1992–2022), during which nutrition surveys were conducted in the Zinder and Maradi regions. In total, seven out of the eight regions (excluding Niamey) reported high levels of SAM in more than half of the years surveyed. In 2015, all regions reported high SAM prevalence, coinciding with multiple disease outbreaks. That year, meningitis cases were reported in four regions (Tahoua, Tillabery, Dosso, and Niamey, accounting for 65% of total cases), while 90% of cases of measles occurred in Zinder, Maradi, and Agadez [46]. Interestingly, there was minimal overlap between years affected by food crises and those with high SAM prevalence. Notable exceptions include 2010, which saw a significant reduction in cereal production, and 1998 and 2000, which were preceded by severe droughts.

On the national level, GAM is significantly correlated with several socioeconomic indicators (Table 2). GAM has a positive correlation with global undernutrition, under-5 mortality, and HIV prevalence, and it is negatively correlated with life expectancy at birth. The prevalence of underweight children is significantly correlated with GAM. These socioeconomic factors are known to be direct consequences (mortality, underweight, and life expectancy) or aggravating factors (HIV prevalence) of malnutrition. However, there are no significant correlations with SAM. Most of these correlations between GAM and socioeconomic indicators are due to synchronous linear trends over time. When the linear trend in the time series is removed, only the prevalence of underweight children remains significantly correlated with GAM (Table 2).

### 3.2. Relationships Between Malnutrition and Rainfall

**Figure 5:**
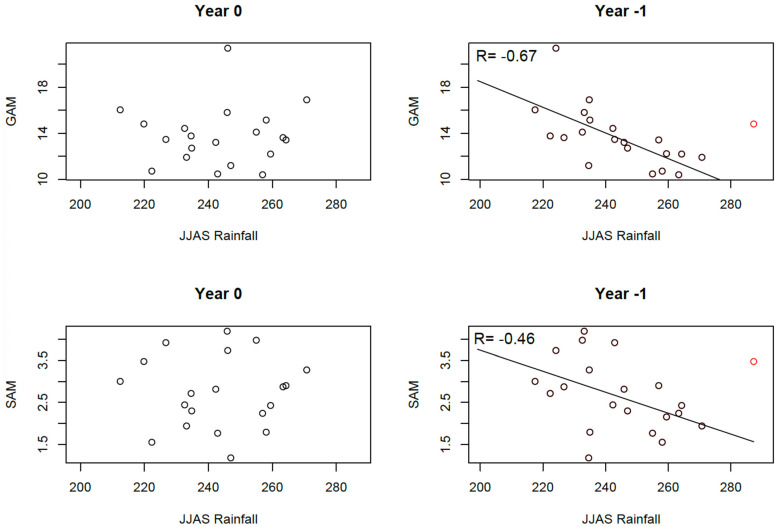
Relationships between malnutrition and summer rainfall at the national level. **Left**: Relationship between Global Acute Malnutrition (GAM) index, Severe Acute Malnutrition (SAM) index, and JJAS rainfall of the year. **Right**: Same but using JJAS rainfall of the year preceding the malnutrition data. All values are averaged across Niger. The dot in red is the rainfall year 1999 and the malnutrition year 2000. The line is drawn from the linear regression between malnutrition and rainfall of the previous year, excluding the rainfall year 1999.

**Figure 6:**
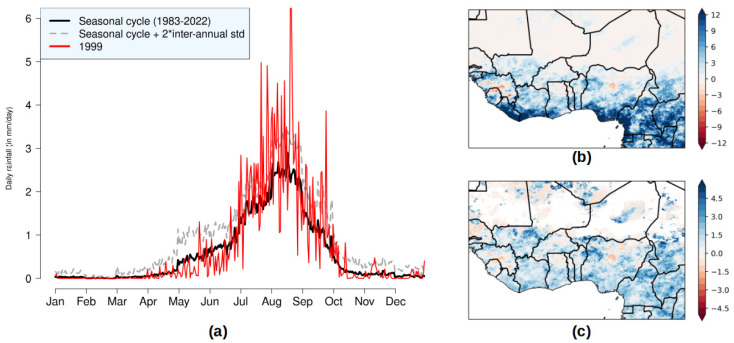
(**a**) Daily precipitation in Niger during 1999 compared to the averaged 1983–2022 seasonal cycle, and the seasonal cycle plus two standard deviations calculated per day over all year. (**b**) Anomalies of the number of heavy rainy days (i.e., days with rainfall ≥ 20 mm) in West Africa in 1999 relative to 1983–1998. (**c**) Same as (**b**) but normalized by the standard deviation over 1983–2022.

On the national scale, no statistical relationship is observed between malnutrition indices (national averages of GAM and SAM derived from the mean of the regional values) and rainfall indices of the same year (Figure 5). However, a linear decrease (increase) in malnutrition is evident when rainfall from the previous year increases (decreases), as depicted in the scatter plots of Figure 5. This lagged relationship can be explained by the impact of summer rainfall on crop production, as annual crops harvested in autumn constitute the primary food source from late in the year until the next harvest. The clearest relationship is observed between JJAS (June-to-September) rainfall and GAM. The correlation coefficient is R= −0.51, which is not significant at the 5% level (Table 3). However, this correlation becomes significant (R = −0.67) when the year 1999 is excluded. The year 1999 was identified as the wettest in the rainfall dataset (red point in Figure 5), with a rainfall anomaly between two and three standard deviations above the mean rainfall. Contrary to expectations, 2000 had a slightly above-normal GAM value instead of a low value. This anomaly could be attributed to political instability following the Nigerien coup d’état in April 1999, which likely disrupted food production and markets. Additionally, devasting floods in 1999 affected over one million people across 11 West African countries, including Sudan [47,48]. Heavy rainfall may have boosted runoff and benefited crop development, but floods likely damaged cultivated areas and reduced production. Analyzing regional rainfall features in 1999 from the TAMSAT dataset supports this hypothesis. Indeed, most of days in July and August in 1999 were characterized by a rainfall amount larger than the seasonal cycle plus twice the inter-annual variability in Niger (Figure 6a). On a larger scale, the number of heavy rainy days in 1999 was larger by up to 10 days in the Sahel relative to 1983–1998 (Figure 6b). We even find local 1999 anomalies larger than 4.5 standard-deviations in Southern Mali, Southwestern and Central Niger, Northwestern Nigeria, and Central Chad (Figure 6c). Despite the clear relationship between GAM and prior-year summer rainfall, no significant correlation is found with other rainfall indices, with temperature indices (Table 3), or with SAM.

**Figure 7:**
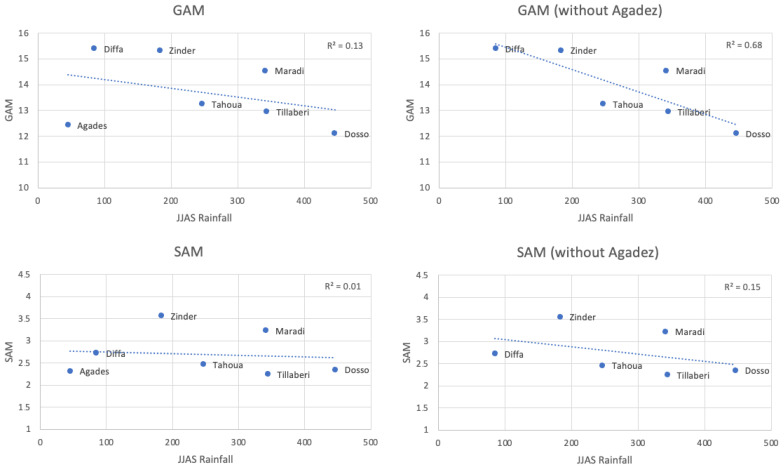
Relationships between the spatial patterns of malnutrition and summer rainfall. Relationship between Global Acute Malnutrition (GAM) index, Severe Acute Malnutrition (SAM) index, and JJAS rainfall of the previous year of 7 of the 8 regions of Niger (Niamey is excluded). Values are multi-year average from 1989 to 2022. The region of Agadez was excluded from the two right panels. The R2 and the dotted line are derived from the linear regression between malnutrition indices and JJAS rainfall of the previous year.

On the regional scale, malnutrition appears to be linked to climatological rainfall (Figure 7). Regions with lower (higher) summer rainfall tend to have higher (lower) malnutrition rates when considering multi-year averages from 1989 to 2022. This relationship is clearer using GAM as the malnutrition proxy rather than SAM and becomes stronger when excluding the Agadez region. Without Agadez, summer rainfall explains 68% of the spatial variance in malnutrition (Figure 7). Agadez’s case is unique; it is the driest region but also among the least affected by malnutrition. This can be attributed to better socioeconomic conditions, reflected in a poverty index more than three times lower than other regions (except Niamey) (Enquête Harmonisée sur le Conditions de Vie des Ménages, 2021–2022). The region relies on diverse livelihood activities, including transhumance, nomadic practices; small-scale vegetable farming [49]; and a low population density, which reduces resource pressure and can limit the size of disease outbreaks, such as cholera and measles/meningitis in 2014, according to WHO health statistics, and 2015 [46]. Agadez has also the lowest annual malaria incidence in Niger [50]. In Niger, malaria infection among children with Severe Acute Malnutrition has been shown to exacerbate undernutrition—particularly by impairing linear growth—even though treating malaria can modestly improve weight gain, underscoring the need for integrated strategies that address both infection control and nutritional rehabilitation in these vulnerable populations [51]. Conservation efforts of oasis ecosystems and forest irrigated agriculture projects since the 1990s, particularly promoting date palm cultivation [52], have likely strengthened the resilience of vulnerable populations.

While summer rainfall drives the regional spatial pattern of malnutrition, year-to-year rainfall variability does not significantly correlate with malnutrition variability at the regional level. Almost no significant correlations are observed between rainfall and malnutrition indices (Table 4) even after excluding 1999, as in Table 3. In addition, no significant correlations were found with temperature indices. This suggests that malnutrition at the regional level is influenced by a complex interplay of local and regional factors, including but not limited to climate. Correlation analyses may fail to isolate the effect of climate, as it is one among many contributing drivers. When averaging spatially (Figure 5) or temporally (Figure 7), local heterogeneities diminish, leaving large-scale forces, such as climate variability, as the primary observable influence.

### 3.3. Relationships Between Malnutrition and Simulated Crop Yields

**Figure 8:**
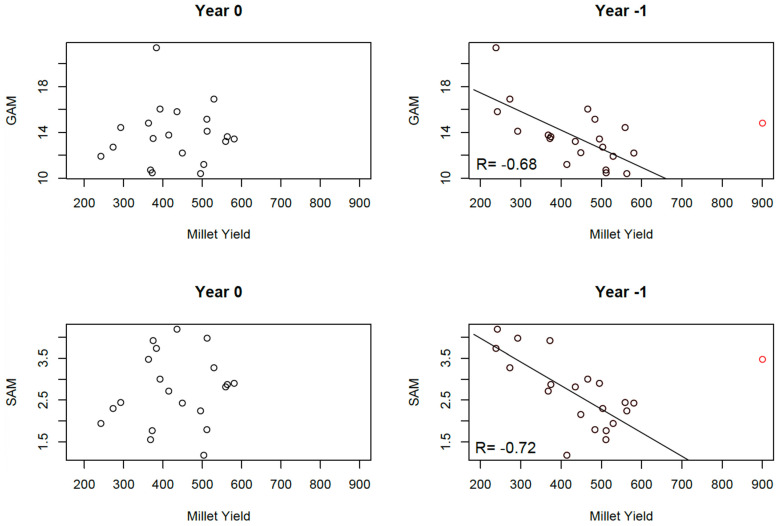
Relationships between malnutrition and simulated crop yields at the national level. **Left**: Relationship between Global Acute Malnutrition (GAM) index, Severe Acute Malnutrition (SAM) index, and simulated crop yield of millet of the year. **Right**: same but using simulated crop yields of the year preceding the malnutrition data. All values are averaged across Niger. The dot in red is the rainfall year 1999 and the malnutrition year 2000. The line is drawn from the linear regression between malnutrition and rainfall of the previous year, excluding the rainfall year 1999.

On the national scale, the relationships between malnutrition and simulated crop yields closely resemble those observed with summer rainfall (Section 3.2). While no statistical relationship is found between malnutrition indices and simulated attainable yield within the same year, clear linear relationships emerge when considering simulated crop yields from the previous year (Figure 8). The year 1999 again appears as an outlier, with crop yields exceeding three standard deviations above normal. Despite this, malnutrition rates (both GAM and SAM) in 2000 were slightly above normal. When excluding this outlier, significant correlations are observed between crop yields and GAM (R = −0.68) and SAM (R = −0.72).

At the regional level, correlations with GAM are stronger when using simulated crop yields rather than rainfall. However, only two regions, Zinder and Tillaberi, exhibit significant correlations at the 5% confidence level (Table 5). In Zinder, the correlation coefficient reaches R = −0.72 with R^2^ = 0.52, indicating that over 50% of the variance in GAM is explained by the variability in crop yields from the previous year. No significant correlations are observed when using SAM as a proxy for malnutrition. Correlations between simulated crop yields and summer rainfall are very high (Table 6), thus complicating the possibility of incorporating both variables into a multiple regression model for predicting malnutrition. Since crop yield simulations integrate rainfall characteristics, including them together in a statistical model could not provide additional predictive value.

## 4. Discussion and Conclusions

This study investigated the statistical links between malnutrition, climate, and simulated crop yields in Niger. While the correlation values are modest, several important insights emerge from this analysis.

Firstly, a statistical link was found between malnutrition and rainfall from the previous year. This relationship does not exist when considering rainfall and malnutrition within the same year but becomes more pronounced when incorporating simulated agricultural yields from the previous year. On the national scale, approximately half of the variance in Global Acute Malnutrition (GAM) is explained by variations in simulated millet yields. This statistical relationship could likely be even stronger since some studies reported successful efforts from Government and International Organizations in reducing malnutrition following agricultural production deficit years [53]. This finding underscores the critical role of the autumn harvest, which builds food stocks largely dependent on the prior year’s summer rains. This time lag highlights the potential for predicting future food crises using rainfall data and/or crop yield simulations, enabling strengthening early warning systems, putting anticipatory action mechanisms in place, and ensuring the timely mobilization of food and nutrition assistance to mitigate malnutrition at least 3 months in advance. It could clearly contribute to the achievement of SDG2 (zero hunger), SDG 3 (better health), and SDG13 (climate adaptation) by anticipating and reducing the impacts of climate variability and change on food security. However, implementing early warning systems and anticipatory action mechanisms is challenging in Niger due to political instability, issues in funding mechanisms, and institutional barriers.

Secondly, the statistical links between malnutrition and rainfall are stronger on the national scale and remain weak or nonexistent on the regional scale. This is also true for simulated crop yields, though correlation values are generally higher. In Zinder, for instance, the correlation between GAM and simulated crop yields reaches R = −0.72 with R^2^ = 0.52, indicating that over half the variance in GAM is explained by crop yield variability. These findings suggest that large-scale climate variability significantly influences malnutrition over the whole of Niger, even if aggregated analyses at the national level fail to capture regional-scale variations. Aggregating regional data helps bypass data heterogeneities and idiosyncratic details, leaving only the influence of large-scale climatic forcing.

Thirdly, no significant correlations were found between malnutrition; temperature indices; and intra-seasonal rainfall indices, such as the onset, end, or length of the rainy season, or the number of dry spells. Only summer rainfall amounts showed correlations with malnutrition at the national level and in some regions, such as Tillaberi, Diffa, and Zinder, though only Tillaberi showed significance at the 5% level. This may be due to the limitations of satellite products such as TAMSAT, which have difficulty reproducing intra-seasonal rainfall characteristics. To check that the problem is not due to the choice of TAMSAT rather than another satellite product, we extracted CHIRPS data in the three regions where the results were clearer with TAMSAT (Zinder, Tillaberi, and Maradi), computed the rainfall indices, and re-performed the correlation analysis. We found similar results, with only JJAS rainfall being significantly correlated with GAM. Ramarohetra et al. [54] found that while satellite-based estimates have large biases in daily rainfall distributions compared to rain gauge data, annual rainfall amounts are better reproduced. However, these biases minimally affect crop yield simulations, as shown in this study. In addition, a single intra-seasonal characteristic may not be sufficient to explain malnutrition. For example, a late onset of rain might be offset by a late end to the rainy season or uninterrupted heavy rains. Crop simulations are therefore more relevant, as they integrate multiple rainfall characteristics that affect crop development. A late onset, early cessation, or reduced rainfall will influence the crop model’s water balance, affecting simulated yields if water stresses occur during critical growth stages. Previous studies using earlier versions of the SARRA-O crop model in Niger demonstrated the impacts of dry spells and monsoon onset on crop yields, depending on the crop’s phenological stage [55]. Simulated crop yields provide a more direct estimation of food availability compared to rainfall indices, thus explaining the stronger correlations between malnutrition indices and simulated yields. These results align with the strategy of the AGRHYMET Regional Center, which uses the SARRA-O crop model within early warning systems to generate food security alerts by combining satellite rainfall estimates with crop simulations [56]. Crop models are likely better suited for investigating the links between climate change and malnutrition than relying solely on rainfall indices. This is particularly relevant given that mean and extreme rainfall in Niger are expected to increase under most climate change scenarios, despite uncertainties regarding the magnitude of these changes [57]. Crop models also account for other critical factors, such as the elevated atmospheric CO^2^ concentrations, which can reduce water stress, and higher temperatures, which may increase water stress and shorten crop cycles [58,59]. Despite these limitations, such intra-seasonal indices may still be valuable for regional studies. For instance, in 2021, the onset of the rainy season was delayed in the Sahelian regions of Niger and Chad, subsequently followed by a brief yet intense wet period and a dry spell in September. The delayed onset and the ensuing dry spell exerted a substantial impact on rainfed crops, with production in Niger and Burkina Faso decreasing by 36% and 10%, respectively, compared with the preceding five-year average. This, in conjunction with other factors, including the ongoing Ukraine war, has led to the onset of food insecurity in several countries, extending from Mali to Chad [60].

Finally, while correlations with GAM and summer rainfall or simulated crop yields were significant at the national level and for some regions, no significant coefficients were found for Severe Acute Malnutrition (SAM) index. The lack of any statistically significant relationship with Severe Acute Malnutrition (SAM) may stem from the acute and multifaceted nature of SAM episodes. Sudden triggers, like conflict, disease outbreaks, or short-term disruptions in food access, often fall outside the scope of region-wide rainfall or yield data. In addition, local healthcare conditions and demographic factors can strongly influence SAM outcomes. For instance, the political crisis in Niger in 2023 and subsequent border closures have prevented the delivery of humanitarian food aid, thus triggering a SAM episode in the context of already high vulnerability due to food shortages and high prices. A more detailed analysis that incorporates socioeconomic, healthcare, and conflict-related indicators would likely provide deeper insights into the drivers of SAM. This suggests that severe malnutrition crises lie in the complex interplay between climate stressors and socioeconomic factors, where climate variability amplifies existing vulnerabilities rather than being an isolated cause. The pathways though which climate change affects food security, water availability, and disease patterns—and, in turn, acute malnutrition—are complex. Extreme weather events, like droughts, floods, and heatwaves, disrupt food production and access, leading to shortages and higher food prices, which exacerbate SAM. However, populations in conflict zones or those with limited access to resources and basic services are particularly vulnerable to both climate change and acute malnutrition. These compounded effects make it challenging to isolate climate change as the primary driver of SAM [61].

Any modelling study has its limitations, and we recognize some caveats in the design of our experiments. This study could be improved using additional indices of malnutrition and rainfall. Here, we only use GAM and SAM, which are measures of the impact of malnutrition, whereas other indices, such as stunting, global chronic malnutrition, and food security indices based on nutrition surveys, could be very relevant to include. In addition, malnutrition data gaps (1993–1995, 1997, 1999, and 2001–2004) can lead to biases in the statistical analysis, and more complete datasets would be valuable for such analyses.

However, this raises the question of the availability of these data to perform such analyses over time at the regional level. Additional climate indices could also be integrated into the analysis. For instance, the list established by Rowell et al. [34] includes 16 climate indices that have been deemed relevant to the agricultural sector. It includes more rainfall indices but also climate metrics based on wind temperatures. However, the additional tests artificially increase the chances to erroneously conclude the presence of a significant correlation with the Bonferroni effect [43].

Another limitation is that the research relies on correlation analysis, and therefore we cannot claim causal relationships between climate variability, crop production, and malnutrition in Niger. There are numerous interacting factors, such as food prices, conflict, economic conditions, healthcare accesses, and aid interventions, which could introduce regional heterogeneities into malnutrition patterns in Niger but also act as confounding factors, affecting the robustness of the findings. To control for confounding in the analyses, we correlate simulated crop yield with 53 development indicators at the national level (Supplementary Table S3). No significant correlations are found except for the prevalence of underweight, which is strongly linked to malnutrition. Supplementary Table S3 suggests that confounding is unlikely at the regional level. However, the control of confounding factors was not possible at the regional level due to the limited availability of subnational socioeconomic data. In addition, the inclusion of subnational socioeconomic data could provide a more holistic view and allow for a more advanced statistical approach, such as multivariate regression, machine learning, or structural equation modelling, and strengthen causal inference.

## Figures and Tables

**Table 2:** Relationships between SAM and GAM and development indicators at the national level. Correlation between SAM and GAM and development indicators. A linear trend was removed for both malnutrition and development indicators in the two last columns. Significant values at the 1% level are shown in bold and frame.

Series Name	GAM	SAM	GAM (Detrended)	SAM (Detrended)
Population, total	−0.52	−0.06	0.12	0.16
Population growth (annual %)	−0.12	−0.07	−0.11	−0.06
Surface area (sq. km)	NA	NA	NA	NA
Population density (people per sq. km of land area)	−0.52	−0.06	0.12	0.16
Poverty headcount ratio at national poverty lines (% of population)	NA	NA	NA	NA
Poverty headcount ratio at USD 2.15 a day (2017 PPP) (% of population)	0.74	−0.09	0.10	−0.66
GNI, Atlas method (current USD)	−0.49	−0.03	0.12	0.18
GNI per capita, Atlas method (current USD)	−0.46	−0.03	−0.01	0.09
GNI, PPP (current USD)	−0.48	−0.08	0.10	0.02
GNI per capita, PPP (current USD)	−0.49	−0.11	0.08	−0.06
Income share held by lowest 20%	−0.76	0.16	−0.73	0.24
**Life expectancy at birth, total (years)**	**−0.55**	−0.09	−0.08	0.01
Fertility rate, total (births per woman)	0.49	0.05	−0.08	−0.09
Adolescent fertility rate (births per 1000 women ages 15–19)	0.41	0.00	−0.16	−0.16
Contraceptive prevalence, any method (% of married women ages 15–49)	−0.47	0.13	−0.14	0.26
Births attended by skilled health staff (% of total)	−0.47	−0.10	0.30	0.25
**Mortality rate, under 5 (per 1000 live births)**	**0.56**	0.18	0.16	0.21
**Prevalence of underweight, weight for age (% of children under 5)**	**0.76**	0.49	**0.64**	0.49
Immunization, measles (% of children ages 12–23 months)	−0.49	0.01	−0.01	0.21
Primary completion rate, total (% of relevant age group)	−0.53	0.00	−0.10	0.19
School enrollment, primary (% gross)	−0.52	−0.02	−0.06	0.17
School enrollment, secondary (% gross)	−0.42	0.13	0.03	0.31
School enrollment, primary and secondary (gross), gender parity index (GPI)	−0.43	0.04	0.43	0.31
**Prevalence of HIV, total (% of population ages 15–49)**	**0.59**	0.12	0.29	0.07
Forest area (sq. km)	0.51	0.13	0.03	0.10
Terrestrial and marine protected areas (% of total territorial area)	0.24	−0.16	0.22	0.03
Annual freshwater withdrawals, total (% of internal resources)	−0.45	−0.03	0.06	0.04
Urban population growth (annual %)	−0.09	0.27	0.29	0.38
Energy use (kg of oil equivalent per capita)	0.26	0.25	0.30	0.24
Electric power consumption (kWh per capita)	0.22	0.32	0.43	0.64
GDP (current USD)	−0.49	−0.07	0.12	0.07
GDP growth (annual %)	0.24	0.01	0.40	0.03
Inflation, GDP deflator (annual %)	0.09	−0.24	0.08	−0.21
Agriculture, forestry, and fishing, value added (% of GDP)	−0.28	−0.48	−0.20	−0.46
Industry (including construction), value added (% of GDP)	0.07	0.34	0.13	0.34
Exports of goods and services (% of GDP)	0.33	0.21	0.14	0.18
Imports of goods and services (% of GDP)	−0.15	0.01	0.09	0.05
Gross capital formation (% of GDP)	−0.42	−0.08	0.07	0.00
Revenue, excluding grants (% of GDP)	NA	NA	NA	NA
Time required to start a business (days)	0.13	−0.42	0.05	0.05
Domestic credit provided by financial sector (% of GDP)	NA	NA	NA	NA
Tax revenue (% of GDP)	NA	NA	NA	NA
Military expenditure (% of GDP)	−0.26	0.31	0.22	0.54
Mobile cellular subscriptions (per 100 people)	−0.40	0.11	0.28	0.42
High-technology exports (% of manufactured exports)	0.16	−0.02	0.14	−0.03
Merchandise trade (% of GDP)	0.00	0.01	0.10	0.02
Net barter terms of trade index (2015 = 100)	−0.46	−0.13	−0.20	−0.12
External debt stocks, total (DOD, current USD)	−0.26	0.02	0.23	0.11
Total debt service (% of exports of goods, services and primary income)	−0.05	−0.15	−0.09	−0.14
Net migration	0.06	0.37	0.22	0.40
Personal remittances, received (current USD)	−0.36	−0.09	0.15	−0.02
Foreign direct investment, net inflows (BoP, current USD)	−0.26	0.00	0.11	0.07
Net official development assistance and official aid received (current USD)	−0.38	−0.04	0.20	0.09

**Table 3:** Relationships between malnutrition and climate indices at the national level. Correlation between Global Acute Malnutrition (GAM) index, Severe Acute Malnutrition (SAM) index, and climate indices of the previous year with and without including the year 1999. Significant values at the 5% level are shown in bold. A linear trend was removed before computing correlation with temperature indices.

GAM	With 1999	Without 1999
	year n − 1	
JJAS rainfall	−0.51	**−0.67**
Onset	−0.28	−0.38
Duration	0.25	0.32
Cessation	−0.11	−0.14
Dry spells	−0.04	−0.10
Tmean	0.30	0.28
Tmin	0.22	0.20
Tmax	0.34	0.32
Tx90p	0.22	0.19
Tn90p	0.27	0.25
Wsdi	−0.14	−0.16
**SAM**	**With 1999**	**Without 1999**
	year n − 1	
JJAS rainfall	−0.27	−0.46
Onset	−0.21	−0.21
Duration	0.35	0.35
Cessation	0.14	0.14
Dry spells	0.16	0.16
Tmean	0.19	0.24
Tmin	0.21	0.26
Tmax	0.16	0.20
Tx90p	0.14	0.22
Tn90p	−0.07	−0.01
Wsdi	−0.29	−0.25

**Table 4:** Relationships between malnutrition and rainfall indices at the regional level. Correlation between Global Acute Malnutrition (GAM) index, Severe Acute Malnutrition (SAM) index, and rainfall indices of the previous year. Significant values at the 5% level are shown in bold. The year 1999 is excluded.

		GAM	SAM
		year n − 1	year n − 1
Zinder	JJAS rainfall	−0.58	−0.20
	Onset	−0.20	0.06
	Duration	0.05	0.01
	Cessation	−0.32	0.13
	Dry spells	0.16	0.39
Maradi	JJAS rainfall	−0.25	−0.14
	Onset	−0.43	−0.17
	Duration	0.33	0.14
	Cessation	−0.27	−0.09
	Dry spells	0.32	0.04
Tillaberi	JJAS rainfall	**−0.60**	−0.37
	Onset	−0.56	−0.23
	Duration	0.46	0.44
	Cessation	−0.12	0.27
	Dry spells	−0.24	−0.36
Tahoua	JJAS rainfall	−0.28	−0.09
	Onset	−0.44	−0.37
	Duration	0.29	0.50
	Cessation	−0.36	0.11
	Dry spells	−0.32	−0.22
Diffa	JJAS rainfall	−0.53	−0.17
	Onset	−0.21	−0.33
	Duration	0.49	0.48
	Cessation	0.53	0.30
	Dry spells	−0.45	−0.20
Dosso	JJAS rainfall	−0.39	−0.11
	Onset	−0.28	0.30
	Duration	0.36	−0.08
	Cessation	0.09	0.39
	Dry spells	0.11	0.36
Agadez	JJAS rainfall	−0.44	−0.33
	Onset	0.20	−0.01
	Duration	−0.13	0.10
	Cessation	−0.08	0.11
	Dry spells	0.20	0.00

**Table 5:** Relationships between malnutrition and simulated crop yields at the regional level. Correlation between Global Acute Malnutrition (GAM) index, Severe Acute Malnutrition (SAM) index, and simulated crop yields of millet of the previous year. Significant values at the 5% level are shown in bold. The year 1999 is excluded.

	GAM	SAM
	year n + 1 (w/o 1999)	year n + 1 (w/o 1999)
Zinder	**−0.72**	−0.51
Maradi	−0.51	−0.28
Tillaberi	**−0.62**	−0.27
Tahoua	−0.45	0.27
Diffa	−0.50	−0.10
Dosso	−0.57	0.27
Agadez	−0.37	−0.37

**Table 6:** Relationships between JJAS rainfall and simulated crop yields at the regional level. Correlation simulated crop yields of millet and JJAS rainfall of the same year. Significant values at the 5% level are shown in bold.

	Synchronous Correlation
Zinder	**0.80**
Maradi	**0.73**
Tillaberi	**0.82**
Tahoua	**0.69**
Diffa	**0.82**
Dosso	**0.74**
Agadez	**0.62**

## Data Availability

The raw data supporting the conclusions of this article will be made available by the authors upon request.

## Supplementary Materials

The following supporting information can be downloaded at https://www.mdpi.com/article/10.3390/ijerph22040551/s1, Table S1: World Development Indicators metadata. Table S2: Parameters used in the SARRA-O crop simulation model. Table S3: Relationships between development indicators and simulated crop yield at the national level. Correlation between development indicators and simulated crop yield of the previous year with and without including the year 1999. Significant values at the 1% level are shown in bold.
